# The Origin of the *Cyathea delgadii* Sternb. Somatic Embryos Is Determined by the Developmental State of Donor Tissue and Mutual Balance of Selected Metabolites

**DOI:** 10.3390/cells10061388

**Published:** 2021-06-04

**Authors:** Anna Mikuła, Wojciech Tomaszewicz, Michał Dziurka, Andrzej Kaźmierczak, Małgorzata Grzyb, Mirosław Sobczak, Piotr Zdańkowski, Jan Rybczyński

**Affiliations:** 1 Center for Biological Diversity Conservation in Powsin—Polish Academy of Sciences Botanical Garden, Prawdziwka 2, 02-973 Warsaw, Poland; w.tomaszewicz@obpan.pl (W.T.); m.grzyb@obpan.pl (M.G.); j.rybczynski@obpan.pl (J.R.); 2 The Franciszek Górski Institute of Plant Physiology, Polish Academy of Sciences, Niezapominajek 21, 30-239 Kraków, Poland; m.dziurka@ifr-pan.edu.pl; 3 Department of Cytophysiology, Faculty of Biology and Environmental Protection, University of Łódź, Pomorska 141/143, 90-236 Łódź, Poland; andrzej.kazmierczak@biol.uni.lodz.pl; 4 Department of Botany, Institute of Biology, Warsaw University of Life Sciences (SGGW), Nowoursynowska 159, 02-787 Warsaw, Poland; miroslaw_sobczak@sggw.edu.pl; 5 Institute of Micromechanics and Photonics, Warsaw University of Technology, Św. Andrzeja Boboli 8, 02-525 Warsaw, Poland; pzdankowski@gmail.com

**Keywords:** amino acids, carbohydrates, cytomorphology, phenolic acids, phytohormones, polyamines, somatic embryogenesis

## Abstract

Somatic embryogenesis is the formation of a plant embryo from a cell other than the product of gametic fusion. The need to recognize the determinants of somatic cell fate has prompted investigations on how endogenous factors of donor tissues can determine the pattern of somatic embryo origin. The undertaking of this study was enabled by the newly developed experimental system of somatic embryogenesis of the tree fern *Cyathea delgadii* Sternb., in which the embryos are produced in hormone-free medium. The contents of 89 endogenous compounds (such as sugars, auxins, cytokinins, gibberellins, stress-related hormones, phenolic acids, polyamines, and amino acids) and cytomorphological features were compared between two types of explants giving rise to somatic embryos of unicellular or multicellular origin. We found that a large content of maltose, 1-kestose, abscisic acid, biologically active gibberellins, and phenolic acids was characteristic for single-cell somatic embryo formation pattern. In contrast, high levels of starch, callose, kinetin riboside, arginine, and ethylene promoted their multicellular origin. Networks for visualization of the relations between studied compounds were constructed based on the data obtained from analyses of a Pearson correlation coefficient heatmap. Our findings present for the first time detailed features of donor tissue that can play an important role in the somatic-to-embryogenic transition and the somatic embryo origin.

## 1. Introduction

In theory, every cell of living tissues can be used to establish an in vitro cell culture and the production of whole plants. However, cells originating from different tissues and distinct parts of the plant body have different regenerative potential. It is well known that cells from seedlings and juvenile-stage plants exhibit strong morphogenic competence [1]. In induced somatic embryogenesis (SE), which is the best example of totipotency expressed in a large number of plant species, the choice of explant is of fundamental importance for the pathway by which somatic embryos are produced (direct or indirect route) and their origin (unicellular or multicellular) [2]. The type of explant used to induce SE also affects the frequency and morphology of somatic embryos [3,4,5]. The understanding of how the type of donor tissues impacts regenerative processes under tissue culture conditions is essential to work out approaches and protocols for improving the effectiveness of in vitro regeneration of plants.

Most probably, the physiological state of the explant is the reason for divergent and specific morphogenic responses of explants subjected to in vitro culture. Plant organs and tissues differ in terms of phytohormone and sugar contents. It has been often reported that their levels change along the axis of such organs as leaf, petiole, and root [6,7,8], and they may differ between the apical and the basal part of cotyledons [9,10] and epicotyls [11]. They also change during the course of organ ontogeny [12]. The interplay and balance between the amounts of phytohormones and sugars can be regulated by the conditions under which plants develop [8,13]. Thus, the plant organs used as initial explants for in vitro cultures may determine their regenerative capacity [7,10,12,14]. Studies on phytohormone status in the leaf, petiole, and root explants of *Populus* spp. showed that the accumulation of zeatin, indole-3-acetic acid, and abscisic acid may have a mediating role during organogenesis [7]. In *Avena sativa*, a negative correlation of endogenous trans-zeatin and kinetin content and a positive correlation of gibberellin A1 (GA_1_) content with haploid embryo formation were revealed [15]. In the case of SE, the acquisition of embryogenic capacity can be regulated by long-term shading of donor plants resulting in their etiolation [13,16]. Dark conditions modify hormonal and carbohydrate profiles, making explants capable of SE [13]. Their disruption contributes to the loss of embryogenic potential [17]. However, among the published studies, there are also those in which a relationship between embryogenic competence and endogenous hormone levels was not found (e.g., [18]). More comprehensive research on physiological status of initial explants is needed to better understand how the type of donor plant material impacts regenerative processes under tissue culture conditions.

Somatic embryogenesis in the tree fern *Cyathea delgadii* Sternb. was first induced by our group 6 years ago [19]. The studies conducted so far indicate that this experimental system, using only etiolated sporophytes as a source of explants capable of SE, can be an important and useful tool in exploring the acquisition of embryogenic competence under external hormone-free conditions [13,20]. The somatic embryos of *C. delgadii* originate from single epidermal cells when stipe explants are taken [19,20], or they are of multicellular origin (from a group of epidermal and cortical cells) in the case of using internode explants [4]. In both pathways, the explant cells are turned into an embryogenic state with high frequency and effectiveness after a short induction period lasting for about 8 or 4 days, respectively [4,19,20]. To date, there is no information on the endogenous compounds that distinguish the initial explants giving rise to somatic embryos in direct SE controlled only by internal factors.

The objective of this study was to explore cytomorphological and biochemical bases underlying the pattern of somatic embryo origin using newly developed experimental system of the tree fern *C. delgadii*.

## 2. Materials and Methods

### 2.1. Plant Material, Culture Conditions, and Evaluation of the SE Efficiency

Primary culture of *Cyathea delgadii* Sternb. was established following the procedure described by Mikuła et al. [19]. In the study, 5-month-old cultures of somatic embryo-derived sporophytes that developed 3 or 4 leaves and were maintained in constant darkness were used as a source of samples. They were cultured in hormone-free medium with half-strength Murashige and Skoog’s [21] macro- and micronutrients (½MS) supplemented with a full complement of vitamins and 2% (*w*/*v*) sucrose, and solidified with 0.7% (*w*/*v*) agar (Duchefa Biochemie, Haarlem, The Netherlands). The pH was adjusted to 5.8 before autoclaving. The cultures of 5 sporophytes per 400 mL jar were maintained in a climatic chamber at 24 ± 1 °C. The explants were derived from the lower part of the youngest leaf stipe and from the internode of stem (in the tree fern *C. delgadii,* the stem grows vertically as a rhizome) between the first and second leaf [4]. The 2.5 mm long fragments of stipes (STs) and 1.5 mm long fragments of internodes (INs) were used for microscopic and chromatographic analyses immediately after their dissection.

Another set of the ST and IN explants was used for assessment of their ability to produce somatic embryos. The explants were cultured in Petri dishes on ½MS medium, the same as above except supplemented with 1% (*w*/*v*) sucrose, in darkness. The efficiency of SE was calculated as percentage of explants developing somatic embryos related to the total number of cultured explants after 2 months of culture. For this purpose, 60 explants per each type of plant material were examined. The experiment was repeated 3 times.

### 2.2. Microscopic Examinations

#### 2.2.1. High-Resolution Optical 3D Microscopy

Images of epidermis surface of initial explants and those under culture were taken with an Alicona Infinite Focus G5 high-resolution optical 3D microscope (Alicona, Graz, Austria). The ImageJ program package was used to measure the length and width of epidermal cells. Numbers of divided cells and non-divided cells in ST and IN explants were counted at the 14th and 6th day of culture, respectively, on 132 randomly selected fields of view. The different times of data collection were due to the fact that the epidermal cells of STs and INs began to divide after 8 and 4 days of culture, respectively [4]. In parallel, the length and width of at least 770 undivided and at least 260 divided epidermal cells per explant type were measured. Thereafter, the length-to-width (L/W) ratio for epidermal cells was calculated.

#### 2.2.2. Light Microscopy

Dissected ST and IN samples were immediately fixed in a mixture of 2.5% (*w*/*v*) paraformaldehyde (Fluka, Buchs, Switzerland) and 2.5% (*v*/*v*) glutaraldehyde (Sigma, Aldrich, St. Louis, MO, USA) [4] and then post-fixed in 2% (*w*/*v*) osmium tetroxide dissolved in 0.05 M sodium cacodylate buffer (Fluka) for 6 h at 4 °C. The samples were progressively dehydrated through a series of ethanol solutions, followed by propylene oxide substitution. They were infiltrated with a graded series of Epon epoxy resin (Sigma-Aldrich, St. Louis, MO, USA) mixtures (for 48 h in total) and the resin was polymerized at 65 °C for 16 h. Semi-thin sections (2.5 µm thick) were taken on an Ultracut E ultramicrotome (Leica, Wetzlar, Germany) and stained for 10 min with 0.1% (*w*/*v*) toluidine blue in 1% (*w*/*v*) borax. After washing with distilled water, they were examined under a Vanox light microscope (Olympus, Tokyo, Japan) equipped with a Sony ILCE-7 digital camera (Sony, Tokyo, Japan) and an Olympus cellSens Standard ver. 1.7 image analysis system (Olympus).

#### 2.2.3. Transmission Electron Microscopy (TEM)

The ultrathin sections (90 nm thick) were cut with a Leica EM UC6 ultramicrotome (Leica) and collected on 100 mesh copper grids. The sections were contrasted in a saturated 50% (*v*/*v*) ethanolic solution of uranyl acetate for 30 min and then in 0.04% (*w*/*v*) lead citrate for another 30 min. Specimens were examined under a FEI 268D ‘Morgagni’ (FEI Corp., Hillsboro, OR, USA) TEM operating at 80 kV and equipped with an Olympus-SIS ‘Morada’ digital camera (Olympus). At least 20 sections from different samples of both explant types were examined.

#### 2.2.4. Environmental Scanning Electron Microscopy (ESEM)

Freshly dissected ST and IN samples were examined under a FEI QUANTA 200 (FEI Corp.), an ESEM microscope operating at 0.75 Tr vacuum and at a relative humidity of up to 100%.

#### 2.2.5. Fluorescence Microscopy

Callose was stained according to method developed by Kaźmierczak [22]. The explants were fixed in 2.5% (*v*/*v*) glutaraldehyde (Sigma-Aldrich) in 0.1 M sodium phosphate buffer (pH 7.4) immediately after their dissection (0 h) and 0.5 and 1.5 h post-dissection. The plant material was double-washed with 0.1 M K_2_HPO_4_ (pH 7.0), then incubated for 2 min at 20 °C and for 15 min at 100 °C in Tris-EDTA (pH 8.5) buffer. The samples were washed twice with 4 mM K_2_HPO_4_ (pH 9.0) for 2 min and stained for 15 min with 0.05% (*w*/*v*) aniline blue (Water blue; Fluka) dissolved in 4 mM K_2_HPO_4_. They were washed 3 times with the same buffer and immediately used to measure callose amounts. Four specimens from ST and IN explants were photographed using a Nikon Optiphot-2 epi-fluorescence microscope (Nikon, Tokyo, Japan) equipped with a Nikon DDX1200 camera (Nikon) and Act-1 image analysis software (Precoptic, Warsaw, Poland). The images were acquired under excitation light of a blue filter (B2A; 390–420 nm). The amount of callose was calculated based on fluorescence intensity in relation to the background fluorescence using the ScnImage System (Scion Corporation) software (open source; http://www.scioncorp.com, accessed on 3 June 2021), expressed in arbitrary units (a.u.) of fluorescence intensity after staining with aniline blue and measured immediately (0 h), and 0.5 and 1.5 h after sample dissection (Supplementary Figure S1).

### 2.3. Profiling of Plant Metabolites

#### 2.3.1. Preparation of Plant Samples

Collected samples of the ST and IN explants were immediately frozen in liquid nitrogen, lyophilized, and then pulverized using zirconium oxide beads (MM400, Retsch, Haan, Germany). This material was used in further analyses.

#### 2.3.2. Soluble Carbohydrates

Sugars were analyzed according to the method by Hura et al. [23]. About 5 mg of lyophilized and homogenized sample was poured with 1 mL of ultrapure water (Option R, Elga, High Wycombe, UK) and incubated with shaking in a MM 400 (Retsch) at 30 Hz for 15 min. After centrifugation for 5 min at 10,000× *g* (Universal 32R centrifuge, Hettich, Tuttlingen, Germany), the supernatant was aliquoted into halves and one part was diluted with acetonitrile 1:1 (*v*/*v*), filtered (0.22 µm nylon membrane; Costar Spin-X, Millipore Sigma, New York, NY, USA), and analyzed by high-performance liquid chromatography (HPLC) for contents of soluble sugars, whereas the second part was used for analyses of fructooligosaccharide contents.

#### 2.3.3. Starch

The content of starch was estimated based on the amount of glucose released after its enzymatic hydrolysis with α-amylase (Sigma-Aldrich) dissolved in 50 mM potassium phosphate buffer (pH 6.9) with the addition of 6.7 mM of NaCl and amyloglucosidase (Sigma-Aldrich) dissolved in 200 mM sodium acetate buffer (pH 4.5). Pellets were double-rinsed with ultrapure water, purified with 350 µL α-amylase solution (0.2 U/sample), and incubated in a boiling water bath for 10 min. After cooling down, 450 µL of amyloglucosidase was added (15 U/sample), and samples were incubated for 1 h at 50 °C. The supernatant was collected, diluted 1:1 (*v*/*v*) with acetonitrile, and analyzed by HPLC for released glucose.

#### 2.3.4. Fructooligosaccharides

The amount of fructooligosaccharides in samples prepared as described above was estimated after enzymatic hydrolysis of sugar extract (overnight at 40 °C) in a mixture of exo- (40 U/sample) and endo-inulinase (2 U/sample) dissolved in 100 mM sodium acetate buffer (pH 4.5) according to the supplier’s specifications (Megazyme, Bray, Ireland). Then, the suspension was diluted 1:1 (*v*/*v*) with acetonitrile, centrifuged, and analyzed by HPLC. The interference of free glucose, fructose, sucrose, raffinose, and kestose was determined by subtracting their equivalents from the total glucose and fructose pool estimated after the enzymatic hydrolysis.

#### 2.3.5. HPLC Analyses of Neutral Sugars

High-performance liquid chromatography analyses of free sugars, starch, and fructooligosaccharide hydrolysates were performed using an Agilent 1200 system (Agilent Technologies, Waldbronn, Germany) coupled to an ESA Coulochem II 5200A electrochemical detector HPLC (ESA, Chelmsford, MA, USA). Separation of soluble sugars (glucose, fructose, sucrose, and other fructans) and starch hydrolysates (glucose) was carried out using an RCX-10; 7 µm; 250 × 4.1 mm column (Hamilton, OH, USA) in gradient mode of 75 mM NaOH solution, and 500 mM sodium acetate in 75 mM NaOH solution [23].

#### 2.3.6. UHPLC-MS/MS Profiling of Plant Hormones and Related Compounds

Ultrahigh-performance liquid chromatography coupled with a tandem mass spectrometry (UHPLC-MS/MS) was used for targeted profiling of compounds related to auxins, cytokinins, gibberellins, ABA, JA, and SA according to Hura et al. [24] with modifications. Briefly, samples of about 2 mg obtained as described above were spiked with the stable isotope-labelled internal standard mixture and triple extracted in an extraction buffer (methanol/H_2_O/formic acid, 15:4:1 (*v*/*v*/*v*)). Pooled supernatant was evaporated under a nitrogen stream (TurboVap LV, Capiler, Marshall Scientific, Hampkinton, MA, USA) and re-dissolved in 3% (*v*/*v*) methanol in 1 M HCOOH. Then, samples were cleaned up on a hybrid SPE (solid-phase extraction, Bond Elut Plexa PCX; Agilent Technologies, Santa Clara, CA, USA) columns. Targeted profiling of phytohormones and related compounds was conducted in multiple reaction monitoring (MRM) mode on an Agilent Infinity 1260 UHPLC system (Agilent Technologies) coupled with 6410 QQQ LC/MS with ESI (ElectroSpray Interface) ion source (Agilent Technologies). The separation was achieved on an Ascentis Express RP-Amide analytical column (2.7 μm, 2.1 mm × 150 mm; Supelco, Bellefonte, PA, USA) in a linear gradient of H_2_O vs. acetonitrile with 0.01% (*v*/*v*) of HCOOH. Further technical details are given in the legend of Supplementary Table S1. A stable isotope-labelled internal standard for phytohormones analyses consisted of [^15^N_4_]DHZ, [^2^H_5_]ZR, [^15^N_4_]Kin, [^2^H_2_]GA_1_, [^2^H_2_]GA_4_, [^2^H_2_]GA_6_, [^2^H_2_]GA_5_, [^2^H_5_]IAA, [^2^H_6_]ABA, [^2^H_4_]SA, and [^2^H_5_]BA (OlChemim, Olomouc, Czech Republic), [^2^H_5_]JA, (CND Isotopes, Quebec, Canada), and [^2^H_5_]OPDA (Cayman Chem. Comp., Ann Arbor, MI, USA).

#### 2.3.7. Phenolic Acids

The content of phenolic acids was estimated according to the modified method of Hura et al. [23]. Samples were extracted as described for phytohormones. After evaporation under a nitrogen stream (TurboVap LV), the residue was reconstituted in 50 µL of 50% (*v*/*v*) methanol in 1 M HCOOH and diluted to 1.2 mL with 1 M HCOOH prior clean-up in Discovery DPA-6S SPE cartridges (1 mL, 50 mg; Supelco). Then, samples were lyophilized again under nitrogen, reconstituted in 250 µL of methanol, and analyzed on an Agilent Infinity 1260 UHPLC with a fluorescence detector (FLD). Phenolics were separated in a Zorbax Eclipse Plus Phenyl-Hexyl 3.5 µm 3.0 mm × 100 mm column (Agilent Technologies) under a linear gradient of 2% (*v*/*v*) formic acid aqueous solution versus methanol. Excitation and emission wavelengths were dynamically changed [25].

#### 2.3.8. Polyamines

The amount of polyamines was estimated according to a modified method by Hura et al. [26]. Samples were extracted as described for phytohormones above. Extracts were evaporated under nitrogen and resuspended in 500 µL of 6% (*v*/*v*) trichloroacetic acid, then 400 µL of polyamines fraction or pure standards (PUT, SPD, SPM, CAD, 1,6-DAH, 1,3-DAP) were mixed with 400 µL dansyl chloride solution (5 mg mL^−1^ in acetone) and 400 μL saturated sodium carbonate solution. Samples were incubated at room temperature overnight. Dansylated polyamines were triple extracted with toluene and the organic fraction was evaporated under nitrogen. The dried residues were dissolved in 50 µL of methanol and analyzed on an Agilent 1200 system with an FLD and Poroshell 120 EC-C18 3.0 × 50 mm 2.7 µm analytical column (Agilent Technologies) under a linear gradient of water and methanol/acetonitrile (2:1; *v*/*v*), both diluted with 1% (*v*/*v*) HCOOH. Detection was conducted at 350 nm excitation and 510 nm emission wavelengths. Quantitation was done based on calibration curves constructed for dansylated polyamine standards.

#### 2.3.9. Amino Acids

The content of amino acids was estimated by means of a liquid chromatography technique employing an on-line pre-column derivatization procedure according to the method of Schuster [27]. Dried samples containing amino acid fraction were obtained as described for phytohormones above. They were resuspended in 100 µL of 0.1 M HCl containing norvaline and sarcosine as internal standards (ca. 250 nmol mL^−1^ each). Primary amino acids were derivatized with *ortho*-phthalaldehyde, whereas secondary amino acids were derivatized with 9-fluorenylmethyl chloroformate following the protocol by Woodward et al. [28].

An Agilent Infinity 1260 UHPLC system with a diode array detector (DAD) and an FLD and Poroshel 120 HPH 3.0 × 100 mm 2.7 µm analytical column were used. The separation was performed under a linear gradient of 10 mM Na_2_HPO_4_ and 10 mM Na_2_B_4_O_7_ (pH 8.2) versus acetonitrile/methanol/H_2_O (45:45:10; *v*/*v*/*v*). Absorbance at 338 nm and 232 nm, and fluorescence emission at 450 nm upon excitation at 230 nm wavelength, were measured. Quantitation was conducted based on calibration curves for pure amino acid standards, considering the recovery of internal standards.

#### 2.3.10. Ethylene

To enable gas sampling from ST and IN samples, 5 explants of each were put into 50 mL Erlenmeyer flasks containing 20 mL of ½MS medium with 1% (*w*/*v*) sucrose (see Section 2.1) and kept in the darkness to stabilize cell response to the explant excision. After that, flasks were tightly closed with caps with the inserted pipette tips connected via flexible tubes to a pump of an SCS56 handheld ethylene analyzer (Storage Control System, Sparta, UK). Readings of ethylene production in ppm were taken 1, 4 and 6 h after sampling (Supplementary Figure S2). The ethylene production was stabilized between 4 and 6 h after explant excision. Therefore, the results obtained after 6 h were used for further analyses.

### 2.4. Statistical Analyses

Data for the contents of compounds and their ratios are presented as the mean ± standard deviation (SD) of 6 independent replicates. One replicate for each type of plant material consisted of about 100 explants (200 mg fresh weight). The compound balance was expressed by the ratios of their total amounts. Seven explants for each type of plant material and for each time point after their dissection were used for the determination of callose fluorescence intensity. Results were expressed as the mean ± SD of 4 thin free-hand cross-sections prepared for each type of samples. Measurements of ethylene production were taken in 10 repetitions for each type of plant material. Statistical analyses were performed using the Student’s *t*-test embedded in Microsoft Excel software. Only the return of *p* < 0.05 was accepted as being statistically significant.

Correlation networks between endogenous compounds in ST and IN explants were constructed based on the data obtained from the strong Pearson correlation analyses performed in R software. The analysis was generated on the mean values of 6 independent replicates. Data were analyzed at a 0.05 probability level. The correlation matrix heatmap was built using the corrplot v0.84 package [29] for RStudio (RStudio 1.3.1056-1) [30]. Correlations were incorporated into the network if the *r*-value was *r* > 0.8 or *r* < −0.8.

## 3. Results

### 3.1. Cytomorphological Differences between Stipe and Internode Explants

**Figure 1:**
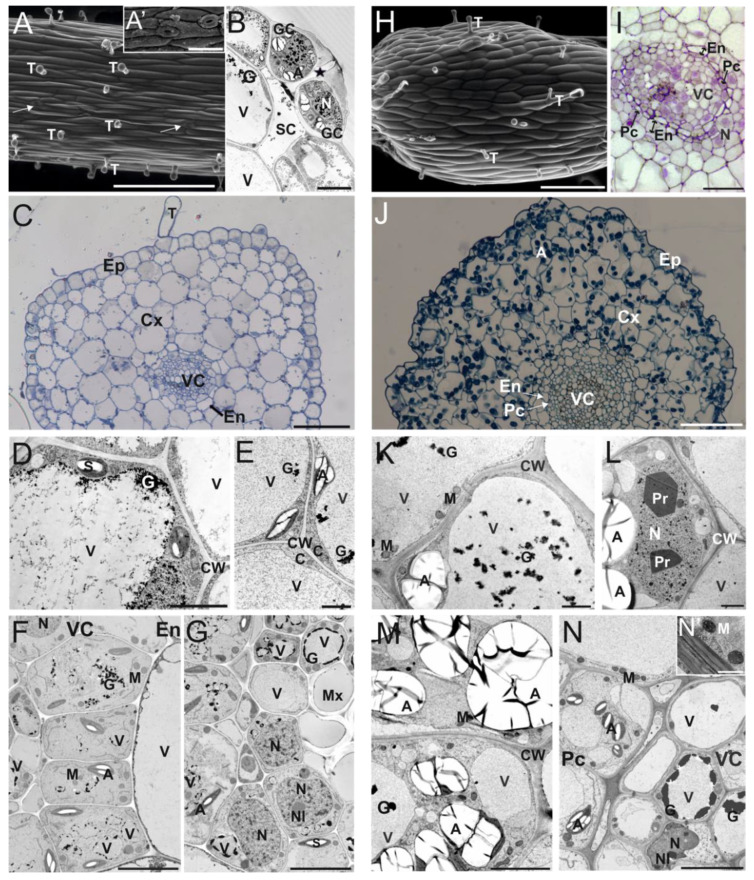
Cytomorphology of stipe (**A**–**G**) and internode (**H**–**N**) explants derived from etiolated in vitro grown sporophytes of *C. delgadii*. (**A**) Scanning electron microscope image of stipe explant with trichomes and stomata (arrows); (**A’**) inset in (**A**): detailed view of stomata. (**B**) Ultrastructure of stoma and guard cells (asterisk indicates stomatal pore). (**C**) Light microscopy image of semi-thin cross-section of a stipe showing 3 different regions: epidermis, 5–6 cell layers of cortex parenchyma with endodermis, and vascular cylinder in the center (stained with toluidine blue). (**B**,**D**–**G**,**K**–**N**) Transmission electron microscopy images of ultrathin sections. (**D**) Ultrastructure of regular epidermal cell. (**E**) Ultrastructure of cortex parenchyma cells containing amyloplasts in a thin layer of cytoplasm. (**F**) Ultrastructure of cortex–vascular cylinder interface. (**G**) Ultrastructure of vascular bundle cells. (**H**) Scanning electron microscope image of internode with trichomes. (**I**,**J**) Light microscopy images of transverse semi-thin sections of an internode (stained with toluidine blue). (**I**) Details of vascular cylinder organization. (**J**) Low-magnification overview showing anatomical zones in internode. (**K**) Vacuolated epidermal cells. (**L**,**M**) Cortex parenchyma cells with paracrystals in nucleoplasm (**L**) and numerous amyloplasts with starch grains in cytoplasm (**M**). (**N**) Ultrastructure of vascular bundle cells. (**N’**) inset in **N**: detailed view of mitochondria and cell wall. Abbreviations: A, amyloplast; C, cytoplasm; CW, cell wall; Cx, cortex; En, endodermis, Ep, epidermis; G, electron-dense vacuolar granules; GC, guard cell; M, mitochondrion; Mx, metaxylem; N, nucleus; Nl, nucleolus; Pc, pericycle; Pr, paracrystal; S, starch; SC, substomatal cavity; T, trichome; V, vacuole; VC, vascular cylinder. Scale bars: (**A**) 300 µm; (**A’**) 50 µm; (**B**) 5 µm; (**C**) 50 µm; (**D**,**E**) 2 µm; (**F**,**G**) 5 µm; (**H**) 250 µm; (**I**) 20 µm; (**J**) 50 µm; (**K**,**L**) 2 µm; (**M**,**N**) 5 µm; (**N’**) 1 µm.

Examinations of the surface of *C. delgadii* ST explants showed that their epidermis was composed of elongated cells, trichomes (Figure 1A), and stomata (Figure 1A,B). Based on the semi-thin sections of STs, 3 anatomical regions were distinguishable: the epidermis, a cortex consisting of 5–6 layers of large parenchymatic cells, and a vascular cylinder (Figure 1C). The epidermal and cortical cells were strongly vacuolated and contained a thin layer of cytoplasm and nucleus located close to the cell wall (Figure 1D,E). The endodermal cells surrounded a centrally located vascular cylinder (Figure 1F,G).

In contrast to the stipes, the epidermis of the IN explants consisted of cells with oblong outlines (Figure 1H). Trichomes were formed, but stomata were absent. Between the epidermis and the vascular cylinder, the 6–8 layers of cortex parenchyma cells with a single-cell layer of endodermis and a vascular cylinder with a pericycle were present (Figure 1I,J). Epidermal cells were flattened in cross-sections and strongly vacuolated, and similarly to cortical cells (but unlike stipe cells), they were rich in amyloplasts containing extremely large starch grains (Figure 1J–M). Numerous electron-dense granules were present inside the vacuoles (Figure 1K,M,N). Paracrystals were sometimes found in the nuclei of cortex parenchyma cells (Figure 1L).

**Figure 2:**
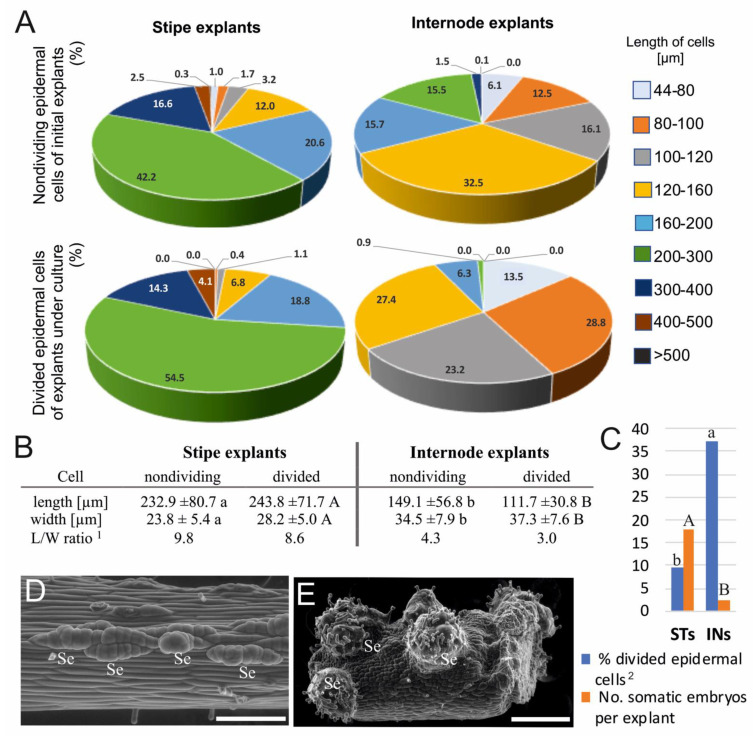
Comparison of biometrical features of epidermal cells, their capacity for division, and the somatic embryo production on stipe and internode explants. (**A**) Percentages of cells in relation to their length. (**B**) Average cell length and width: nondividing epidermal cells of initial explants; divided epidermal cells of explants under culture. (**C**) The ability of the explant cells to divide and produce somatic embryos. (**D**,**E**) Scanning electron microscope images of somatic embryos developed from the single epidermal cells of the stipe explant (**D**) and from multiple adjacent cells of internode explant (**E**). More details about the somatic embryo development were provided by Grzyb and Mikuła [4]. Abbreviations: INs, internode explants; Se, somatic embryo; STs, stipe explants. Scale bars: (**D**) 200 µm; (**E**) 500 µm. ^1^ L/W ratio was calculated as an average ratio between length and width for each cell. ^2^ Percentage of divided epidermal cells in relation to all cells of epidermis determined at 14th (stipes) and 6th (internodes) day of culture.

More than 82% of the measured epidermal cells of the initial ST explants were longer than 160 µm, whereas almost 70% of the IN cells were shorter than 160 µm (Figure 2A). The average length and width of ST cells was equal to 232.9 and 23.8 µm, respectively (Figure 2B). Epidermal cells of IN explants were about 84 µm shorter and more than 10 µm wider than ST cells. The length/width ratio of the ST epidermal cells was more than twice than that of the INs.

In the ST explants, the length of about 90% of all divided cells ranged between 160 and 400 µm, whereas in the case of IN explants, it was in the range between 44 and 160 µm (Figure 2A**)**. The average lengths of the divided epidermal cells were 243.8 and 111.7 µm for ST and IN explants, respectively (Figure 2B). Divided epidermal cells were approximately 3–4 µm wider compared to the cells of initial explants, and their L/W ratio was almost 3-fold greater in STs (8.6) than in INs (3.0). Only 9.5% of epidermal cells of the STs were divided, whereas in the INs, the portion of divided cells reached 37.1% (Figure 2C). The achieved efficiency of SE after 2 months of culture reached 17.83 and 2.45 for the ST and IN explants, respectively. Somatic embryos developed from the single epidermal cells of the ST explants (Figure 2D) and from several adjacent cells of the IN explants (Figure 2E).

The Student’s *t*-test was used to indicate statistical differences between the control explants of the STs and INs (lowercase letters), and between divided cells of both cultured explants (uppercase letters). Data followed by different letters were significantly different at *p* < 0.05.

### 3.2. Differences in the Content of Endogenous Compounds

The 89 detected endogenous compounds were sorted into the specific groups according to their biochemical and physiological roles, namely: carbohydrates, auxins, cytokinins, gibberellins, stress-related hormones, phenolic acids, polyamines, and amino acids. The contents of 65 of them were significantly greater in ST than in IN explants.

**Figure 3:**
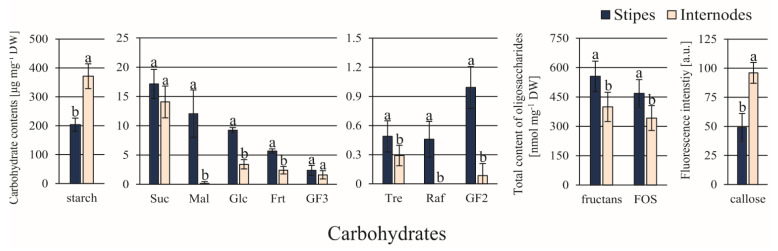
Production of ethylene by the *C. delgadii* stipe and internode explants at 1, 4, and 6 h after sample dissection. The production was stabilized between 4 and 6 h after explant excision. Therefore, the results obtained after 6 h were used for further analyses. Values represent the means ± standard deviation (SD) of 6 independent replicates. The Student’s *t*-test was used to estimate statistical significance of results. Data followed by different letters (a, b) were significantly different at *p* < 0.05.

Among 10 carbohydrates detected, the ST explants contained significantly greater amounts of Mal (about 75-fold), Glc, Frt, Tre, Raf, and GF2 (11.5-fold) compared to IN explants (Figure 3). Only the contents of starch and callose were greater (almost 2-fold) in IN than in ST explants.

**Figure 4:**
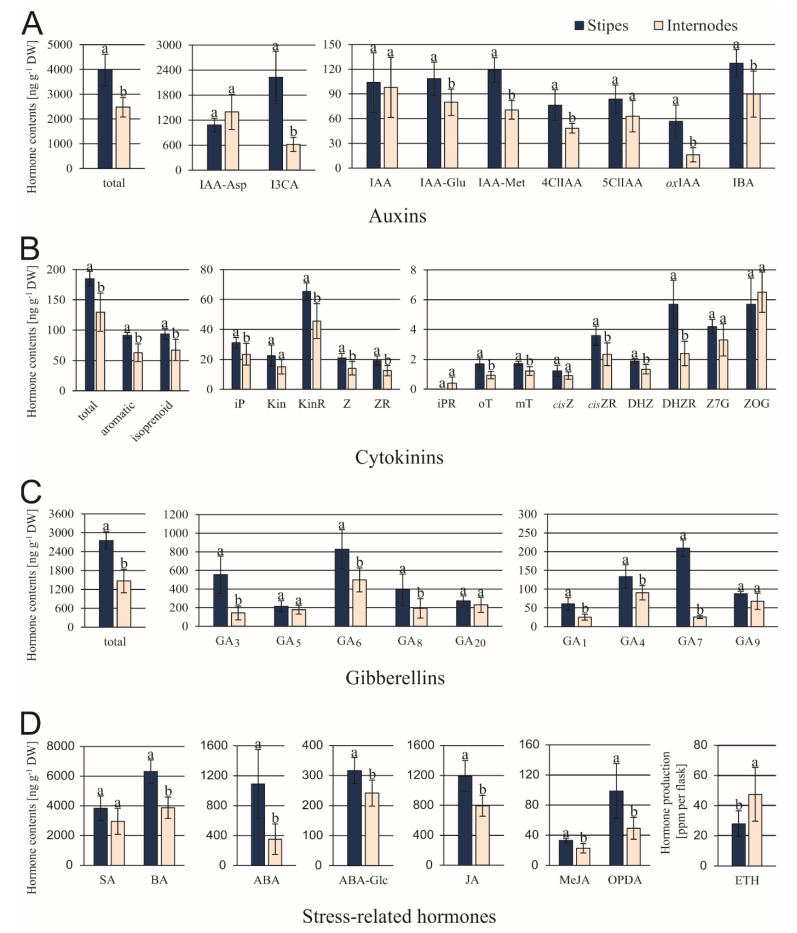
Contents of endogenous auxins (**A**), cytokinins (**B**), gibberellins (**C**), and stress-related hormones (**D**) in *C. delgadii* stipe and internode explants. Values represent the means ± standard deviation (SD) of 6 independent replicates. The Student’s *t*-test was used to estimate statistical significance of results for each type of compounds. Data followed by different letters (a, b) were significantly different at *p* < 0.05.

The ST explants contained significantly greater amounts of 6 out of 9 tested auxins (Figure 4A), but these differences were particularly distinctive in the content of I3CA and *ox*IAA. Among cytokinins, the DHZR accumulated in STs in about 2.9-fold larger quantities compared to INs (Figure 4B). Differences between contents of other cytokinins varied from 1.3- up to 1.8-fold. In both explant types, the KinR amount was greater than any other individual cytokinin. The level of GA_6_ was the greatest among 9 gibberellins examined in both types of explants (Figure 4C). Among bioactive gibberellins (GA_1_, GA_3_, GA_4_, GA_5_, GA_6_, and GA_7_), the most significant differences were found for GA_3_ and GA_7_. Their contents were 3.9- and 4.9-fold greater in ST than in IN explants. The SA and its precursor BA were detected in large amounts in comparison to other stress-related hormones analyzed (Figure 4D). The ST explants contained about 2.4- and 2-fold greater amounts of ABA + ABA-Glc and OPDA (JA precursor), respectively, in comparison to IN ones. Among this group of hormones, only the ethylene production was higher in IN than in ST samples.

**Figure 5:**
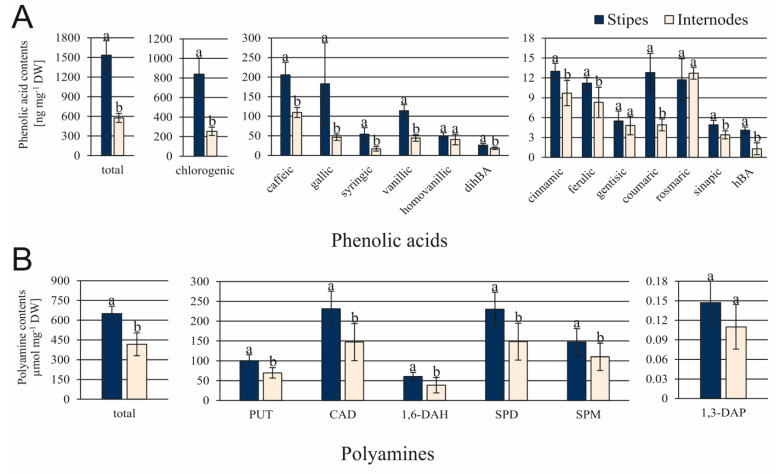
Contents of phenolic acids (**A**) and polyamines (**B**) in stipe and internode explants of *C. delgadii*. Values represent the means ± standard deviation (SD) of 6 independent replicates. The Student’s *t*-test was used to estimate statistical significance of results for each type of compounds. Data followed by different letters (a, b) were significantly different at *p* < 0.05.

The total content of phenolic acids was about 2.7-fold greater in ST in comparison to IN explants (Figure 5A). The most significant differences were found for amounts of chlorogenic, gallic, syringic, vanillic, coumaric, and hBA acids. Their levels were more than 2.5-fold greater in ST than IN explants. Among polyamines, CAD and SPD were the most abundantly produced in both types of explants (Figure 5B). Accumulation of 5 polyamines (PUT, CAD, 1,6-DAH, SPD, and SPM) was significantly greater in ST than in IN explants.

**Figure 6:**
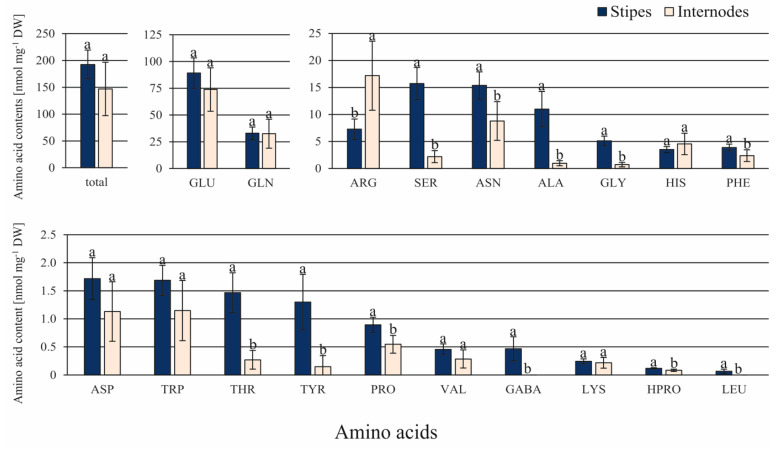
Contents of amino acids in the *C. delgadii* stipe and internode explants. Values represent the means ± standard deviation (SD) of 6 independent replicates. The Student’s *t*-test was used to estimate statistical significance of results for each amino acid. Data followed by different letters (a, b) were significantly different at *p* < 0.05.

Although the contents of 11 amino acids out of 22 tested ones were significantly greater in ST than in IN explants, their total content did not differ significantly (Figure 6). The amounts of ALA, GLY, and SER were between 11.3- and 7.1-fold greater in ST than in IN explants. GABA and LEU were only detected in ST samples, but their amounts were less than 0.5 nmol mg^−1^ DW. Only the amount of ARG was 2.4-fold greater in IN than in ST explants.

Despite significant differences in the total contents of different compounds, the ratios between most of them were similar (Supplementary Table S2). The most significant differences were found in ratios between the amount of various groups of compounds and ethylene or phenolic acids.

### 3.3. Analysis of Relationships between Compounds in Stipe and Internode Explants

**Figure 7:**
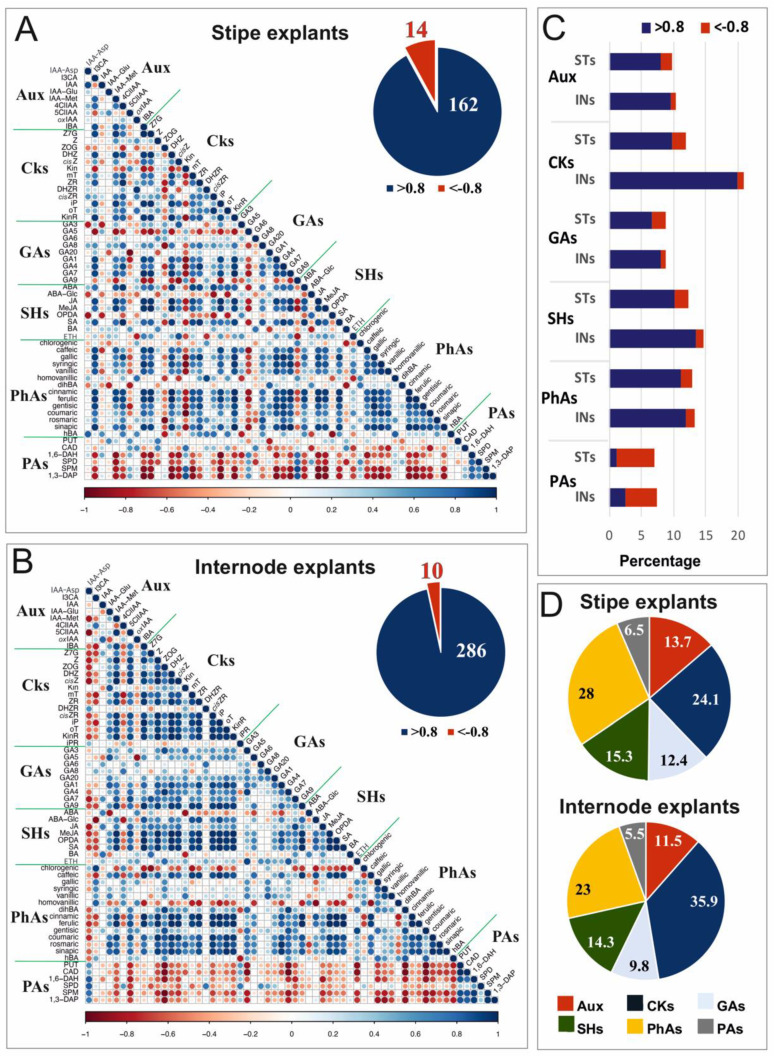
Pearson correlation matrix of the recorded relationships between contents of selected endogenous compounds in stipe (**A**) and internode (**B**) explants (*n* = 6), and quantitative comparison of correlations recorded between them as a result of the Pearson’s correlation matrix analysis, with *r* > 0.8 and *r* < −0.8 (**C**,**D**). (**A**,**B**) The degree of pairwise correlation with respect to the Pearson correlation coefficients is displayed by the color gradient. Positive correlations are displayed in shades of blue, whereas negative correlations are given in shades of red. The corresponding pie chart shows the numbers of negative correlations (*r* < −0.8, in red) compared to numbers of positive correlations (*r* > 0.8, in blue) within the correlation matrices. (**C)** Comparison of percentages of the total numbers of positive (blue) and negative (red) correlations occurring within individual groups of compounds. (**D**) Percentage shares of correlations recorded between the groups of compounds. Abbreviations: Aux, auxins; CKs, cytokinins; GAs, gibberellins; INs, internode explants; PAs, polyamines; PhAs, phenolic acids; STs, stipe explants; SHs, stress hormones.

Pearson correlation coefficients demonstrated significant relationships between individual phytohormones and metabolic compounds such as phenolic acids or polyamines. The relationships were illustrated in the correlation matrices that were separately constructed for ST (Figure 7A) and IN (Figure 7B) explants. The compounds had 14 negative correlations at the probability level of 0.05; i.e., with *r* < −0.8 in the ST explants, compared to 10 negative correlations found for the IN explants and 162 positive correlations with *r* > 0.8 compared to 286, respectively.

The positive and negative correlations within the individual classes of phytohormones and metabolic substances were quantitatively similar in both explant types, excluding cytokinins, for which the very strong correlations (with a coefficient value between ±0.8 and ±1) occurred almost twice as often in IN samples in comparison to ST ones (Figure 7C). There was a set of 175 statistically significant (above 0.8) correlations among cytokinins (21%), and only 8 of them were negative. In both explant types, polyamines were the only group of compounds in which negative correlations were 3–4 times more numerous than positive ones (Figure 7C). The percentage share of the recorded correlations between the groups of compounds showed that the correlations between cytokinins or phenolic acids and other compounds dominated in both types of explants (Figure 7D).

## 4. Discussion

### 4.1. Effect of Cell Size on Embryogenic Competence

One of the factors that clearly differentiated external appearance of the *C. delgadii* explants was the length and the width of epidermal cells. Cell size is not rigidly fixed, but varies strongly within a plant regarding the tissue type and its developmental stage. Depending on the ratio of surface area to volume of the cells, the cytoplasm/nucleus ratio changes, and it affects the rate of nutrient uptake, protein concentrations, and transcription frequency, as well as the physiological state in general [31]. Cell size is often indicated as one of the key parameters affecting embryogenic competence. Many authors maintain that small cells with electron-dense cytoplasm are able to rapidly divide and thus form primary units for somatic embryo development [31,32,33]. Fransz and Schel [32] showed that the cells of the abaxial scutellar region of the *Zea mays* embryo start to divide rapidly, first forming embryogenic callus, and then discrete clusters of small cytoplasm-rich cells that differentiate into somatic embryos of single-cell origin. Cells from the adaxial side of scutellum differentiated only into large and more vacuolated parenchymatous cells unable to develop into somatic embryos. A similar behavior of scutellum epithelial cells was also shown in rice and orchardgrass [33,34]. Sometimes, epidermal or subepidermal cells of initial explants undergo several divisions giving rise to a meristematic zone composed of several cell layers, where somatic embryos then develop [35,36]. The cells involved in SE usually contain a large nucleus with a conspicuous nucleolus, electron-dense cytoplasm with numerous ribosomes, mitochondria, amyloplasts, and short profiles of the rough endoplasmic reticulum. However, such characteristics seem to be typical for distinguishing between embryogenic and non-embryogenic cells in indirect SE. Our research has shown that despite strong vacuolation, the cells of both explant types produced somatic embryos via direct SE. Thus, in donor tissues, the embryogenic competence is not only restricted to cells with obvious ultrastructural features of meristematic cells. Considering the cell size, an L/W ratio closer to 1 than 10 seems to determine the multicellular pathway of somatic embryo induction. However, additional stressful treatment can easily reverse the pattern of SE [4]. Thus, the competence of cells to produce somatic embryos was not restricted to a particular type of cells distinguished on the basis of their morphology, and it was consistent with the data published by Toonen and De Vries [37].

### 4.2. Relationship between Carbohydrates and Somatic Embryo Origin

Carbohydrate metabolism has profound effects on plant growth, particularly on cell divisions and expansion. Sugars can also act as signaling molecules and gene-expression regulators [38]. Among them, starch, a storage polysaccharide, plays an important role in somatic embryo induction and development as a source of energy and carbon, and as an osmotic regulation agent [39]. Starch deposition frequently accompanies tissue proliferation [40,41] and callus development [42]. It leads to the formation of the starch-rich high mitotic activity zone, in which the embryogenic zone located at the submarginal region is then initiated. Large amounts of starch were found in embryogenic calli in contrast to nearby non-embryogenic cells [43,44,45]. In *C. delgadii*, starch accumulated in amyloplasts of IN explants appears to be an excessive source of energy for rapid divisions of many epidermal and cortical cells, and thus for the development of somatic embryos from more than one cell [4]. In contrast, the relatively low content of starch in ST explants combined with sucrose added to the culture medium is sufficient to only activate the divisions of single epidermal cells [19]. The accumulation of great numbers of starch grains in plastids is also interpreted as the first observable step toward the acquisition of an embryogenesis-competent state. Recently, it has been shown that a lipid transfer protein driving the totipotency of somatic cells in cotton specifically accumulates on the amyloplast membranes [46]. Dedifferentiated cells containing plastids without starch grains undergo symmetrical cell divisions that lead only to unorganized cell proliferation and non-embryonic callus formation. This provides new insights in a better understanding of the role of amyloplasts and starch grains in the identification of cells having embryogenic abilities. In IN explants of *C. delgadii*, a high level of starch accumulation can therefore be considered as an important factor determining the multicellular origin of somatic embryos. A similar conclusion can be also drawn in the case of callose deposition. This polysaccharide plays important roles in plant development and response to multiple stresses [47]. In ferns, callose is involved in the differentiation and function of stomatal complexes [48]; however, its small content in ST explants combined with the presence of numerous stomata does not indicate a link with this function. Callose also accompanies the formation and maturation of a cell plate preceding the formation of new cell wall during cell division [49]. The role of callose has been documented in the morphogenesis of young *Anemia phyllitidis* gametophytes [22]. Great content of callose in IN explants can be explained by the greater number of cell divisions that frequently occur in smaller and more meristematic cells.

Analysis of Raf, Mal, GF2, Glc, and Frt contents revealed that these soluble carbohydrates were predominantly present in ST samples. The trisaccharide Raf, along with Suc, Glc, and Frt, is one of the metabolites that is the most significantly correlated with freezing tolerance [50]. It is synthesized from the sucrose in the cytosol and accumulates in plastids/chloroplasts during plants’ exposure to cold, showing a protective effect on thylakoid membranes and photosynthetic machinery. It is also involved in desiccation tolerance of somatic embryos [51]. In light of these findings, the difference in Raf content seemed to be unrelated to the origin of somatic embryos. The disaccharide Mal is a reducing sugar that is produced during starch hydrolysis. The exogenously applied Mal supports the growth and development of fern gametophytes [52], stimulates a strong accumulation of endogenous Mal [53], and promotes SE in some tissue-culture systems of spermatophytes [53,54]. We, therefore, assumed that the level of endogenous Mal can be a marker for predicting the pattern of somatic embryo initiation.

The trisaccharide GF2 is the simplest inulin-type fructan and the main product of sucrose–sucrose fructosyltransferase [55]. It is the key element for the generation of a set of oligofructans that play different protective roles in plant responses to divergent stresses. Oligofructans, mainly stored in the cell sap (the osmoregulatory compartment of the plant cell), can help to maintain the proper osmotic balance during initiation of somatic embryos from single cells. It is confirmed by the size of epidermal cells of ST explants (the length of more than 82% of them exceeds 160 µm) and a high level of their vacuolation, as well as high content of GF2.

### 4.3. Relationship between Phytohormone and Other Compounds Status versus Somatic Embryo Origin

Auxins and cytokinins act synergistically to regulate cell division and antagonistically to control the formation of buds and roots [56]. Although the importance of the balance between them for initiation of morphogenetic response in tissue culture is well known [6], their constant ratio observed in both types of *C. delgadii* explants (Supplementary Table S2) did not indicate their influence on the initiation of somatic embryos. The total content of auxins was greater in ST than in IN explants, mainly due to unequal distribution of auxin and cytokinin; i.e., I3CA and KinR. Tryptophan-derived indolic metabolites, including I3CA, are the major group of compounds accumulating in response to pathogen infection or abiotic stress [57]. However, the involvement of the I3CA in SE is still not clarified. The strong correlations (with *r* > 0.8) between cytokinins and other compounds were found, which, in the IN explants, were about twice as high as in the ST samples (Figure 4). They were related to physiologically active cytokinins (Z, *cis*Z, iP, and oT), as well as to other forms of cytokinins (KinR, ZOG, DHZ, ZR, and *cis*ZR). KinR in the ST samples correlated positively with Z7G, DHZ, ZR, iP, and mT levels, whereas in IN explants, it was positively correlated with the majority of cytokinins. These results suggested that KinR controls homeostasis between storage and physiologically active forms of cytokinins, and probably can activate divisions of appropriate cells and control the rate of cell dedifferentiation in explants. Moreover, the activity of ZR, a generally known cytokinin receptor ligand [58], is also involved in nitrogen signaling, symplasmic communication, xylem transport, and control of morphogenetic pathways leading to somatic embryo formation. It also showed that *cis*Z and *cis*ZR are important in micropropagation and controlling of plant developmental stages by delaying senescence-related changes [59]. This role was confirmed by our experiments with SE in *C. delgadii*, because during one month of culture, the dying cells were not observed (data not shown). Consequently, it could be assumed that the interrelationship between KinR and active forms of cytokinins may be one of the general key factors triggering both pathways of SE.

Gibberellins, the class of phytohormones that stimulate cell divisions by inducing changes in primary cell wall and DNA synthesis [22], are also implicated in SE. Our results indicated that small concentrations of gibberellins favored the multicellular origin of somatic embryos. The bioactive GA_3_ and GA_7_ seemed to be equally important for the unicellular origin of somatic embryos, as their amounts were much greater in ST explants than in IN ones. These gibberellins are mainly located in growing plant organs [60], and their greater content in the ST samples seemed to be related to the developmental status of this part of the fern sporophyte, being more heterogeneous in their rate of specialization than INs. Analyses revealed 25 negative correlations between gibberellins and cytokinins in the ST explants compared to only two in the IN samples (Figure 7A,B). Several of them were related to physiologically inactive GA_9_ and physiologically active DHZ, *cis*Z, mT, iP, and KinR (or their storage form Z7G). A set of five other negative correlations was found between Kin and GA_1_, GA_4_, GA_7_, GA_8_, and GA_20_. This suggested that Kin may stimulate induction of SE by inhibition of the above-enumerated gibberellins. Moreover, disabling of GA_5_ and GA_9_, and simultaneous activation of IAA-Asp and I3CA, can be of vital importance for the establishment of the unicellular somatic embryo induction pathway.

In the case of stress-related hormones, our results showed a significant difference in the content of ABA and its major gluco-conjugate ABA-Glc between the explants. ABA-Glc exhibited weak or no biological activity. It appears to be a traffic form of ABA, and serves as storage product that can be hydrolyzed to release free ABA [61]. Unlike ABA-Glc, ABA is a key hormone regulating the final phases of somatic embryo development [62], as well as the embryogenic response of in vitro cultured tissues of many plant species [14,63,64], including *C. delgadii* [13]. In the fern, high content of ABA completely inhibited SE in the ST explants derived from non-etiolated donor plantlets [13]. In STs of etiolated plantlets, ABA may be responsible for the low frequency of cell divisions, which are limited to 18 single epidermal cells per explant, on average [4]. ABA is necessary to control the activity of stomata [65], which are present in epidermis of *C. delgadii* STs, but absent in INs. Therefore, a certain level of endogenous ABA, which is directly linked to the developmental status of distinct plant organs, may determine the single-cell origin of somatic embryos.

Positive correlations between ABA and the most of cytokinins revealed in the *C. delgadii* ST explants, in opposition to negative correlations in IN explants, suggested that cell divisions preceding unicellular initiation of somatic embryos remained under the control of ABA. This effect can be enhanced by ethylene, whose decreased biosynthesis is required for local expression of genes encoding flavin monooxygenases involved in local biosynthesis and subsequent distribution of auxin [66]. The SA and BA, being considered as factors associated with pathological processes [67], are regarded as regulators of phenolic acid levels and activity. Their content appeared to be 2.7-fold greater in ST than in IN explants, mainly due to unequal distribution of chlorogenic, caffeic, and gallic acids. According to [68], the treatment with exogenous SA considerably increases the level of chlorogenic acid biosynthesis, leading to a reduction in the ability of explants to produce embryos. Likewise, Reis et al. [69] demonstrated the adverse effect of caffeic and gallic acids on SE. On the contrary, a strong positive relationship between somatic embryo induction, development, and separation from the mother tissue through the formation of a separation zone consisting of phenolics-rich cells was shown using microscopic tools [69,70]. Phenolic compounds can disturb endogenous auxin levels, affecting the activity of IAA oxidase [71] and promoting somatic embryo induction. However, in general, phenolic acids are considered as detrimental compounds during in vitro culture, since their exudation and oxidation negatively affect the explants due to browning [72]. Our analyses indicated that high levels of endogenous phenolic compounds favored the single-cell origin of somatic embryos, whereas their low levels were related to an induction of embryos of multicellular origin.

Polyamines show species-, organ-, and tissue-specific distribution patterns inside the plant body. They are usually accumulated in greater amounts in the generative organs, in contrast to small amounts found in vegetative ones (leaf, stem, root) [9]. In *C. delgadii*, they appeared more abundantly in stipes than in internodes. Numerous reports have shown the positive correlation between the content of endogenous polyamines (i.e., PUT, SPD, or SPM) in donor organs and their embryogenic capacity [9,73]. Although the precise mode of action of polyamines is not completely understood yet, it seems that they can modulate embryogenic processes along the plant axis by maintaining a balance between them and other compounds. For example, high levels of PUT and SPD did not induce accumulation of IAA and gibberellins [74]. In the *C. delgadii* explants, statistical analyses revealed that the strongest negative correlations were related to this group of compounds, and they were more numerous in the IN than in the ST explants. This can be accepted as a feature characterizing the multicellular origin of somatic embryos.

Among all analyzed amino acids, the significant difference in the abundance of ARG in both types of the *C. delgadii* explants additionally confirmed the importance of the physiological state of donor tissue for the pathway of somatic embryo formation. ARG is a precursor of polyamines. It has the highest nitrogen-to-carbon ratio, which makes it especially suitable for storage of organic nitrogen [75]. However, a relationship between greater production of ARG and the content of polyamines has not been confirmed yet. Recent reports indicated that ARG metabolism has a pivotal role in the regulation of gametophore shoot formation in the moss *Physcomitrium patens* [76]. The multicellular pattern of somatic embryo initiation may require an extreme accumulation of nitrogen for the synthesis of nucleic acids and proteins necessary for intensive divisions. Additional research in this area should be undertaken to explain the involvement of amino acids in the regulation of SE.

## 5. Conclusions

Analysis of the current results in light of our previous achievements led to the conclusion that the ability of explants to produce somatic embryos is not restricted to a particular type of cells distinguished on the basis of their morphology or ultrastructure. The pattern of the somatic embryo origin depends on the developmental status of the plant organ that has been used as an explant source, and is correlated with physiological state of donor tissues.

Taken altogether, we concluded that the following features of *C. delgadii* explants are crucial to enter the single-cell somatic embryo formation pathway:

(1) High contents of ABA, biologically active gibberellins, and phenolic acids (indicate a more advanced developmental status of explant in which only a limited number of cells is competent to enter embryogenic pathway);
(2) High content of Mal and GF2 (help to maintain the osmotic homeostasis in strongly vacuolated cells);
(3) Numerous positive correlations between ABA and cytokinins (suggest that cell divisions preceding unicellular pathway of somatic embryo formation are controlled by the former).

On the other hand, the following features seemed to be triggering for entering the multicellular pathway of somatic embryo production:

(1) High contents of storage substances (as starch providing carbon backbones and energy and ARG as a source of organic nitrogen, which facilitate quick and extensive cell divisions);
(2) High content of callose (indicates a more meristematic character of the explant cells);
(3) Increased ethylene production (involved in local induction of auxin biosynthesis).

The fold changes of selected endogenous compounds that can be imperative for origin of somatic embryos by unicellular or multicellular pathway in ST and IN explants of *C. delgadii* are summarized in Table 1.

Our comprehensive study provides new insights for the importance of donor plant material in the pattern of somatic embryo origin, and is helpful in a better understanding of the associations between the physiological states of donor organs and pathways of SE.

## Figures and Tables

**Table 1:** Differences in selected metabolites that can be imperative for origin of somatic embryos by the unicellular or multicellular pathways.

Metabolite	Stipes	Internodes
**Carbohydrates**	/-fold greater/	
Starch		1.9
Callose		1.7
Mal	75.0	
GF2	11.0	
**Hormones**		
GA_3_	3.9	
GA_7_	4.9	
ABA	3.1	
ETH		1.7
**Others**		
Total phenolic acids	2.7	
ARG		2.4

## Data Availability

The data sets generated for this study are available on request from the corresponding author.

## Supplementary Materials

The following are available online at https://www.mdpi.com/article/10.3390/cells10061388/s1, Figure S1. The intensity of callose fluorescence in *C. delgadii* stipe and internode explants. The relative amount of callose was expressed in arbitrary units (a.u.) of fluorescence intensity after staining with aniline blue and measured immediately and 0.5 and 1.5 h after sample dissection. The Student’s *t*-test was used to estimate statistical significance of results. Data are presented as the mean ± standard deviation (SD) from 7 samples (each consisting of 4 thin free-hand cross sections). Data indicated with different letters were significantly different at *p* < 0.05. Table S1. Multiple reaction monitoring (MRM) transitions for the analyzed plant hormones and other compounds in positive ion mode (+ESI), capillary voltage 4 kV, gas temperature 350 °C, gas flow 12 l min^−1^, and nebulizer pressure 35 psi. MassHunter software was used to control the LC-MS/MS system and in data analysis. For MRM parameters optimization, the MassHunter Optimizer was used. Compound functioning as internal standard (ISTD) were labelled with stable isotopes (D-^2^H and N15-^15^N). Figure S2: Production of ethylene by the *C. delgadii* stipe and internode explants at 1, 4, and 6 h after sample dissection. The production was stabilized between 4 and 6 h after explant excision. Therefore, the results obtained after 6 h were used for further analyses. Values represent the means ± standard deviation (SD) of 6 independent replicates. The Student’s *t*-test was used to estimate statistical significance of results. Data followed by different letters were significantly different at *p* < 0.05. Table S2. Ratios of total contents of different compounds sorted into the basic classes in stipe and internode explants of *C. delgadii*.

## Abbreviations

1,3-DAP	1,3-Diaminopropane
1,6-DAH	1,6-Diaminohexane
4ClIAA	4-Chloroindole-3-acetic-acid
5ClIAA	5-Chloroindole-3-acetic acid
ABA	Abscisic acid
ABA-Glc	Abscisic acid glucosyl ester
ALA	Alanine
ARG	Arginine
ASN	Asparagine
ASP	Aspartic acid
AVA	5-Aminovaleric acid
BA	Benzoic acid
CAD	Cadaverine
*cis*Z	*cis*-Zeatin
*cis*ZR	*cis*-Zeatin riboside
DHZ	Dihydrozeatin
DHZR	Dihydrozeatin riboside
dihBA	3,4-Dihydroxobenzoic acid
DW	Dry weight
ETH	Ethylene
FOS	Fructooligosaccharides
Frt	Fructose
GABA	Gamma-aminobutyric acid
GA	Gibberellin
GF2	1-Kestose
GF3	Nystose
GF4	Fructosylnystose
Glc	Glucose
GLN	Glutamine
GLU	Glutamic acid
GLY	Glycine
hBA	p-Hydroxobenzoic acid
HIS	Histidine
HPRO	*trans*-4-Hydroxyproline
I3CA	Indole-3-carboxylic acid
IAA	Indole-3-acetic acid
IAA-Asp	Indole-3-acetyl-l-aspartic acid
IAA-Glu	Indole-3-acetyl-l-glutamic acid
IAA-Met	Indole-3-acetic acid methyl ester
IBA	Indole-3-butyric acid
ILE	Isoleucine
IN	Internode
iP	N6-Isopentenyladenine
iPR	N6-Isopentenyladenosine
JA	Jasmonic acid
Kin	Kinetin
KinR	Kinetin riboside
LEU	Leucine
LYS	Lysine
Mal	Maltose
MeJA	Jasmonic acid methyl ester
MET	Methionine
MS	Murashige and Skoog’s medium
mT	*meta*-Topolin
OPDA	12-Oxo-phytodienoic acid
oT	*ortho*-Topolin
*ox*IAA	Oxindole-3-acetic acid
PHE	Phenylalanine
PRO	Proline
PUT	Putrescine
Raf	Raffinose
SA	Salicylic acid
SE	Somatic embryogenesis
SER	Serine
SPD	Spermidine
SPM	Spermine
ST	Stipe
Suc	Sucrose
THR	Threonine
Tre	Trehalose
TRP	Tryptophan
TYR	Tyrosine
VAL	Valine
Z	*trans*-Zeatin
Z7G	*trans*-Zeatin-7-glucoside
ZOG	*trans*-Zeatin-o-glucoside
ZR	*trans*-Zeatin riboside
