# Supplementary material for: The Origin of the Cyathea delgadii Sternb. Somatic Embryos Is Determined by the Developmental State of Donor Tissue and Mutual Balance of Selected Metabolites
Source: Cells. 2021 Jun 4;10(6):1388. doi: 10.3390/cells10061388 (PMC8229038; doi:10.3390/cells10061388)
Supplement: Supplementary file 1 [file cells-10-01388-s001.zip › cells-1227157-supplementary.pdf]

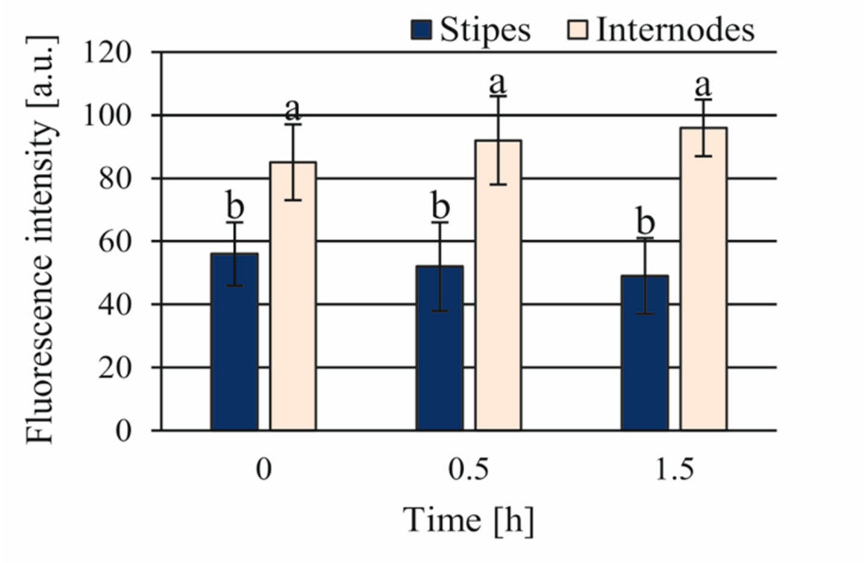

Supplementary Figure S1. The intensity of callose fluorescence in *C. delgadii* stipe and internode explants. The relative amount of callose was expressed in arbitrary units (a.u.) of fluorescence intensity after staining with aniline blue and measured immediately, 0.5 and 1.5 h after sample dissection. The Student's test was used to estimate statistical significance of results. Data are presented as the mean ± standard deviation (SD) from 7 samples (each consisting of 4 thin free-hand cross sections). Data indicated with different letters are significantly different at $P < 0.05$.

Supplementary Table S1. Multiple reactions monitoring (MRM) transitions for the analysed plant hormones and other compounds at positive ion mode (+ESI), capillary voltage 4 kV, gas temperature 350 °C, gas flow 12 l min^{-1}, and nebulizer pressure 35 psi. MassHunter software was used to control the LC-MS/MS system and in data analysis. For MRM parameters optimization MassHunter Optimizer was used. Compound functioning as internal standard (ISTD) were labelled with stable isotopes (D - ^{2}H and N15 - ^{15}N).

Compound		Type of ion	Quantifier transition (precursor/product ions) (*m/z*)	Fragmentor voltage (V)	Collision energy (V)	Retention time (min)
ABA		$[M-H_2O+H]^+$	247.4/187.2	80	14	17.67
D-ABA	ISTD	$[M-H_2O+H]^+$	253.4/191.3	80	14	17.59
ABA-Glc		$[M-H_2O+H]^+$	409.2/247.1	104	14	12.24
BA		$[M+H]^+$	1124.1/78.0	60	14	9.05
D-BA	ISTD	$[M+H]^+$	128.1/84.2	60	14	8.48
*cis*Z		$[M+H]^+$	220.2/136.3	85	9	2.75
*cis*ZR		$[M+H]^+$	352.2/220.3	120	9	6.67
D-OPDA	ISTD	$[M+H]^+$	270.3/252.2	84	5	23.72
DHZ		$[M+H]^+$	222.2/136.0	124	18	2.35
N15-DHZ	ISTD	$[M+H]^+$	226.2/140.0	124	18	2.32
DHZR		$[M+H]^+$	354,2/222,1	124	14	6.26
GA1		$[M-H_2O+H]^+$	331.3/285.3	100	14	11.32
D-GA1	ISTD	$[M-H_2O+H]^+$	333.3/287.2	58	9	11.27
GA3		$[M-H_2O+H]^+$	329.3/311.3	100	14	10.89
GA4		$[M-H_2O+H]^+$	315.3/269.3	100	14	21.80
D-GA4	ISTD	$[M-H_2O+H]^+$	317.3/271.2	88	9	21.78
GA5		$[M-H_2O+H]^+$	285.1/115.0	96	5	18.07
D-GA5	ISTD	$[M-H_2O+H]^+$	287.3/115.0	96	5	18.05
GA6		$[M-H_2O+H]^+$	329.3/115.1	104	14	13.69
D-GA6	ISTD	$[M-H_2O+H]^+$	331.3/115.1	96	5	13.65
GA7		$[M-H_2O+H]^+$	313.2/223.1	104	14	21.58
GA8		$[M-H_2O+H]^+$	319.3/257.2	102	9	5.41
I3CA		$[M+H]^+$	162.2/118.1	58	9	10.89
IAA		$[M+H]^+$	176.1/130.3	51	9	10.91
D-IAA	ISTD	$[M+H]^+$	181.1/135.1	38	14	10.63
IAA-Asp		$[M+H]^+$	291.2/130.1	54	25	5.09
IAA-Glu		$[M+H]^+$	305.2/130.1	58	29	9.89
IBA		$[M+H]^+$	204.1/186.4	69	9	19.64
iP		$[M+H]^+$	204.1/148.3	90	9	7.37
iPR		$[M+H]^+$	336.2/204.1	124	14	13.50
JA		$[M+H]^+$	211.3/151.2	80	14	19.20
D-JA	ISTD	$[M+H]^+$	216.3/153.2	80	5	19.14
Kin		$[M+H]^+$	216.1/188.3	90	9	4.86
N15-Kin	ISTD	$[M+H]^+$	220.1/192.3	90	9	4.82
KinR		$[M+H]^+$	348.2/216.3	116	9	9.21
MeJA		$[M+H]^+$	225.3/151.2	58	5	22.51
OPDA		$[M+H]^+$	293.3/275.2	68	9	25.54
*ox*IAA		$[M+H]^+$	192.2/146.1	54	9	5.34
SA		$[M+H]^+$	139.2/121.2	80	14	11.93
D-SA	ISTD	$[M+H]^+$	143.2/125.2	80	14	11.60
Z		$[M+H]^+$	220.2/136.3	85	9	2.29
Z7G		$[M+H]^+$	382.1/220.1	122	17	1.92
ZOG		$[M+H]^+$	382.1/202.1	142	17	2.30
ZR		$[M+H]^+$	352.2/220.3	120	9	6.00
D-ZR	ISTD	$[M+H]^+$	357.3/225.2	116	17	5.90

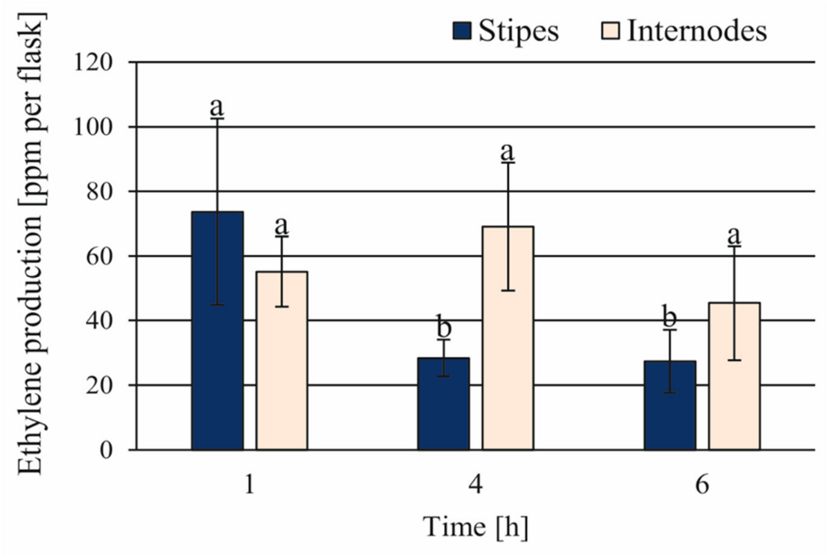

Supplementary Figure S2. Production of ethylene by the *C. delgadii* stipe and internode explants at 1, 4 and 6 h after sample dissection. The production was stabilized between 4 and 6 h after explant excision. Therefore, the results obtained after 6 h were used for further analyses. Values represent the means ± standard deviation (SD) of 6 independent replicates. The Student's test was used to estimate statistical significance of results. Data followed by different letters are significantly different at $P < 0.05$.

Supplementary Table S2. Ratios of total contents of different compounds sorted into the basic classes in stipe and internode explants of *C. delgadii*.

Compound ratios	Stipe	Internode	Stipe/Internode compound ratio
Aux/CKs	21.5 ±2.5a	20.0 ±5.6a	1.0
Aux/GAs	1.5 ±0.2a	1.8 ±0.5a	0.8
Aux/SHs:			
Aux/SA+BA	0.4 ±0.1a	0.4 ±0.1a	1.0
Aux/ABA+ABA-Glc	3.0 ±0.7a	4.5 ±1.6a	0.7
Aux/JA+MeJA+OPDA	3 ±0.2a	3.0 ±1.0a	1.0
**Aux/ETH**	**157.2 ±45.1a**	**62.2 ±28.0b**	**2.5**
**Aux/PhAs**	**2.6 ±0.3a**	**4.3 ±0.7b**	**0.6**
Aux/PAs	6.2 ±1.3a	6.1 ±1.2a	1.0
**CKs/GAs**	**0.070 ±0.005a**	**0.09 ±0.02b**	**0.8**
CKs/SHs:			
CKs/SA+BA	0.02 ±0.002a	0.02 ±0.003a	1.0
**CKs/ABA+ABA-Glc**	**0.1 ±0.04a**	**0.2 ±0.09b**	**0.5**
CKs/JA+MeJA+OPDA	0.1 ±0.02a	0.2 ±0.03a	0.5
**CKs/ETH**	**7.4 ±2.1a**	**3.1 ±1.0b**	**2.4**
**CKs/PhAs**	**0.1 ±0.02a**	**0.2 ±0.06b**	**0.5**
CKs/PAs	0.3 ±0.03a	0.3 ±0.1a	1.0
GAs/SHs:			
**GAs/SA+BA**	**0.3 ±0.03a**	**0.2 ±0.05b**	**1.5**
GAs/ABA+ABA-Glc	2.1 ±0.6a	2.7 ±1a	0.8
**GAs/JA+MeJA+OPDA**	**2.1 ±0.2a**	**1.7 ±0.4b**	**1.2**
**GAs/ETH**	**108.6 ±29.8a**	**34.8 ±12.7b**	**3.1**
**GAs/PhAs**	**1.8 ±0.3a**	**2.5 ±0.6b**	**0.7**
GAs/PAs	4.3 ±0.7a	3.7 ±1.3a	1.2
PhAs/SHs:			
**PhAs/SA+BA**	**0.2 ±0.04a**	**0.1 ±0.02b**	**2.0**
PhAs/ABA+ABA-Glc	1.2 ±0.4a	1.0 ±0.3a	1.2
**PhAs/JA+MeJA+OPDA**	**1.2 ±0.2a**	**0.7 ±0.2b**	**1.7**
**PhAs/ETH**	**61.5 ±21.9a**	**14.3 ±5.9b**	**4.3**
**PhAs/PAs**	**2.4 ±0.5a**	**1.5 ±0.4b**	**1.6**
PAs /SHs:			
PAs/SA+BA	0.07 ±0.01a	0.06 ±0.02a	1.2
**PAs/ABA+ABA-Glc**	**0.5 ±0.2a**	**0.8 ±0.3b**	**0.6**
PAs/JA+MeJA+OPDA	0.5 ±0.1a	0.5 ±0.2a	1.0
**PAs/ETH**	**26 ±8.9a**	**11 ±6.1b**	**2.4**

Values represent the means ±SD of 6 independent replicates. The Student's test was used to estimate significance of results for each type of compounds. Data followed by different letters are significantly different at $P < 0.05$ and shown in bold.
